# Unusual Localization of an Emergent Bacterium, *Raoultella ornithinolytica*

**DOI:** 10.1155/2020/1710271

**Published:** 2020-03-30

**Authors:** Michele Cavaliere, Guido Bartoletti, Pasquale Capriglione, Antonella Miriam Di Lullo, Gaetano Motta, Maurizio Iengo, Elena Cantone

**Affiliations:** ^1^Department of Neuroscience, Reproductive and Odontostomatological Sciences—ENT Section, University “Federico II”, Naples, Italy; ^2^Otorhinolaryngology, Head and Neck Surgery Unit, Department of Mental and Physical Health and Preventive Medicine, Università degli Studi della Campania Luigi Vanvitelli, Naples, Italy

## Abstract

*Raoultella ornithinolytica* is a bacterium belonging to the family *Enterobacteriacae*. It is a rare but emergent cause of human pathologies especially in immunocompromised patients. We described the first case in the literature of isolated external otitis sustained by *Raoultella ornithinolytica* in an immunocompetent host. A 54-year-old Caucasian man with a history of previous myringoplasty came to our attention reporting otalgia and otorrhea. We performed right ear swab for culture examination, meanwhile we started empirical therapy with topic administration of neomycin, without any clinical improvement. The cultural examination showed the presence of a *Raoultella ornithinolytica* infection. After ten days of treatment with oral ciprofloxacin and topic levofloxacin, there was the complete resolution of pain and inflammation. *Raoultella ornithinolytica* must be taken into consideration as an emergent cause of human infection, also in case of external otitis. Infection can be severe and can occur both in immunocompromised and in immunocompetent hosts. Culture test is mandatory to choose the proper therapy and avoid potential severe complications.

## 1. Introduction

*Raoultella ornithinolytica* is a gram-negative, capsulate, aerobic, and nonmotile bacterium belonging to the family *Enterobacteriacae*. Three species of *Raoultella*, initially classified as *Klebsiella,* exist, *R*. *electrica*, *R*. *planticola*, and *R*. *terrigena* [1, 2]. These bacteria have been isolated from aquatic environments, fish, and ticks, where it can produce histamine because of histidine decarboxylase enzyme and can contaminate food, especially not well conserved fish and pork [3]. Histamine toxicity produces symptoms that include flushing of skin, headache, pruritus, and abdominal cramping [3]. This rare but emergent bacterium can cause a wide spectrum of clinical manifestations. For instance, human infections caused by *R*. *ornithinolytica* are rare but can be responsible for bacteraemia, especially in patient with malignancies and immunodeficiencies [4, 5]. Furthermore, *R*. *ornithinolytica* is responsible for urinary, bile tract, tracheal, bronchial, and lungs infections in patients with immunodeficiency. The majority of cases reported in the literature are community acquired infections, especially nosocomial [6]. Neonatal sepsis is rare; however, it can be severe if not treated [7], as well as septic arthritis of the temporomandibular joint. In a case report after an early but temporary response to the antibiotic therapy, this bacterium leads to a complete demolition of the articulation [8]. *R*. *ornithinolytica* not only expresses *β*-lactamase, which provides resistance to commonly used *β*-lactam antibiotics but can also acquire genes for multi-drug resistance [8, 9].

Only a few ear, nose, and throat cases complaining difficulty in swallowing, pain in the throat, rhinosinusitis, and dysphonia are described in the literature. We described the first case in the literature of isolated external otitis (EO) sustained by *R*. *ornithinolytica* in an immunocompetent host.

## 2. Case Presentation

A 54-year-old Caucasian man presented to our hospital with a 7-day history of right otalgia and purulent otorrhea without any systemic symptoms or fever. In order to control pain, the patient was previously treated with acetaminophen alone for 3 days.

There was no significant family, social, or medical history except for a myringoplasty for tympanic membrane perforation of the right ear performed 7 years earlier and a septoplasty performed 5 years earlier.

On physical examination, the patient's blood pressure was found to be 125/70 mmHg, pulse was 80 beats per minute, temperature was 36°C, and respiratory rate was 16 breaths per minute.

We observed pain on tragus pressure, whereas no paralysis of cranial nerves was observed. The right outer ear canal contained purulence with significant hyperemia and edema of skin. Tympanic membrane was impossible to visualize due to the narrowing of the outer ear canal. The rest of the head and neck examination, including the left ear, was normal.

Laboratory data at admission revealed a hemoglobin level of 14 g/dl and white blood cell count of 14.700 per microliter with 70% neutrophils. Inflammation markers were high: erythrocyte sedimentation rate (ESR) was 25 mm/h (normal range 0–22 mm/h) in the first hour, and the level of C-reactive protein (CRP) was 15 mg/L (normal range < 3.0 mg/L).

**Figure 1:**
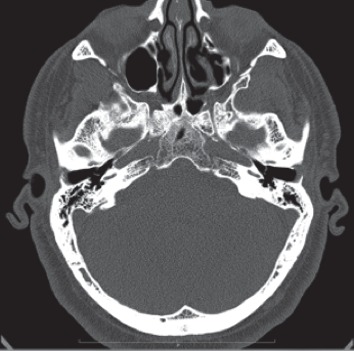
The computed tomography (CT) did not show bone alterations or changes suggestive of malignant external otitis.

The computed tomography scan did not show soft alterations nor mastoid, skull base, and bony changes suggestive of malignant external otitis (Figure 1). We performed right ear swab for culture examination; meanwhile, we started empirical therapy with topic administration of neomycin for 7 days, without any clinical improvement.

The cultural examination showed the presence of a *R*. *ornithinolytica* infection. *R*. *ornithinolytica* isolates were identified with MALDI-TOF MS [10].

On the seventh day, as soon as the susceptibility profile of R. *ornithinolytica* was available (Table 1), we started a systemic therapy with ciprofloxacin 500 mg twice a day for 10 days, and topic therapy using 5 ear drops composed by 3% boric acid in 70% alcohol and 5 drops of levofloxacin twice a day for 10 days, after that we observed a complete resolution of symptoms. In addition, the right ear otomicroscopy showed a dry cavity with only a small granulation on the upper anterior quadrant of the tympanic membrane that disappeared after ten more days of boric acid drops. The six months follow-up did not show recurrence.

## 3. Discussion

We describe a case of culture-confirmed *R*. *ornithinolytica* external otitis, defined as diffuse inflammation of the external ear canal, which may also involve the pinna or tympanic membrane, in an immunocompetent man. *R*. *ornithinolytica* is a rare gram-negative aerobic bacillus belonging to the *Enterobacteriaceae* family. It represents an emergent cause of human infections. Virulence factors involved in the pathogenicity of *R*. *ornithinolytica* are its ability to adhere to human tissues converting histidine to histamine and to form biofilms [11–14]. Generally, *R*. *ornithinolytica* infections are observed in patients with diabetes, immunodepression, or oncological diseases [15]. These infections can cause sepsis, arthritis, urinary tract, and throat impairments; they can be severe and rarely occur in an immunocompetent host [11]. There are only a few cases of EO caused by *R*. *ornithinolytica* reported in the literature, but no one isolated [11]. To our knowledge, this is the first case described of isolated external otitis sustained by *R*. *ornithinolytica* in an immunocompetent host. All in all, *R*. *ornithinolytica* is not considered a virulent pathogen *per se*, but its ability to develop antibiotic resistance can cause severe complications [4, 7, 16]. Hence, the need is to identify the type of bacterium in order to set up the most appropriate therapeutic protocol. This is precisely what we did in our case, especially in the light of the failure of the initially attempted empirical therapy with neomycin. The reported case, based on the susceptibility profile, presented multi-drug resistance (Table 1).

Moreover, in our patient, we did not find any correlation with previous myringoplasty surgery.

The American Academy of Otolaryngology–Head and Neck Surgery Foundation (AAO-HNSF) developed a clinical practice guideline recommending the use of topical preparations for initial therapy of diffuse, uncomplicated EO, whereas systemic antimicrobial therapy should be used in case of extension outside of the ear canal or in the presence of risk factors like diabetes, prior radiotherapy, or immune compromise [17]. However, in our case report we started a systemic therapy based on antimicrobial susceptibility for the lack of therapeutic response to topical therapy.

In our opinion, the culture test is mandatory to choose the proper therapy and to avoid potential severe complications as sepsis, arthritis, and meningitis. Indeed, systemic therapy prevents spreading of the infection, whereas topic therapy medicates the local district. We also recommend toilette of outer ear canal before starting and during the treatment, to provide higher efficacy of the local treatment.

In conclusion, otologists should take into consideration the infection sustained by this bacterium because they can be severe and can occur not only in immunocompromised patients but also, although rarely, in an immunocompetent host.

In our opinion, physicians should be aware of the high rates of antimicrobial resistance of R. *ornithinolytica* as demonstrated by antimicrobial susceptibility in our case report. Further studies have to be done to understand the entity of the diffusion of this bacterium.

## Figures and Tables

**Table 1:** Susceptibility of *R. ornithinolytica*.

Susceptibility of *R. ornithinolytica*		
Antimicrobial	MIC	Interpretation
Ampicillin	≥32	*R*
Amoxicillin/clavulanic acid	≥32	*R*
Piperacillin/tazobactam	>8	*R*
Gentamicin	≤1	*S*
Amikacin	>16	*R*
Nalidixic acid	>8	*R*
Ciprofloxacin	≤0.25	*S*
Fosfomycin	64	*R*
Cefoxitin	≥64	*R*
Cefixime	>16	*R*
Ceftazidime	>16	*R*
Ceftriaxone	>32	*R*
*Trimethoprim/sulfamethoxazole*	>*32*	*R*

MIC: minimum inhibitory concentration.
